# Paediatric Dental Pain Management: Children’s and Parents’ Perspectives in Kosovo

**DOI:** 10.3390/dj14060362

**Published:** 2026-06-11

**Authors:** Fehim Haliti, Shaip Krasniqi, Naim Haliti, Blana Krasniqi, Dion Haliti, Elena Hajdari, Dea Haliti

**Affiliations:** 1 Faculty of Medicine, University of Prishtina, University Dental Clinical Center of Kosovo, 10000 Prishtine, Kosovo; fehim.haliti@uni-pr.edu; 2 Faculty of Medicine, University of Prishtina, University Clinical Center of Kosova, 10000 Prishtine, Kosovo; shaip.krasniqi@uni-pr.edu; 3 Faculty of Medicine, University of Prishtina, Institute of Forensic Medicine, 10000 Prishtine, Kosovo; 4 Faculty of Medicine, Medical University of Tirana, 1005 Tirana, Albania; blana2616@gmail.com; 5 Faculty of Medicine, University of Prishtina, 10000 Prishtine, Kosovo; dionnhaliti@gmail.com (D.H.); elenahajdari@gmail.com (E.H.); deahalitii13@gmail.com (D.H.)

**Keywords:** dental ethics, dental pain, children’s experiences, informed consent, parental perceptions

## Abstract

**Introduction:** The ethics of dental care in children are complex and involve the dentist’s approach, the child’s perception, and the parents’ attitudes. Aim: This study aims to analyse children’s experiences, parents’ perceptions, and the dentist’s approach to dental pain in relation to ethical principles, communication, and informed consent. **Methodology:** This cross-sectional study included 116 paediatric patients, using a semi-structured questionnaire. We assessed demographic data, children’s overall experiences and pain during dental visits, parent–dentist communication, and parents’ ethical approaches to pain management in paediatric patients. **Results:** The majority of children were informed by their parents about the dental visit (97.4%) and the reason for the visit (94.8%). Communication between the dentist and child during the procedure was the main factor that helped reduce fear (61.2%). Most children reported no pain (45.8%) or only minimal pain (51.8%), with needle puncture being the most commonly reported source of dental pain. Overall, parents demonstrated positive ethical attitudes towards paediatric dental care, high satisfaction with dental services (96.6%), and strong support for the development of legal and written guidelines for pain management in children. **Discussion:** The ethical framework for children’s pain management in our dental services is well established, resulting in a high level of satisfaction with the care provided and strong trust in dental professionals. **Conclusions:** This study provides a basis for the promotion of ethical standards for dental procedures in pain management in paediatric patients, and other measures to reduce anxiety and fear should be part of future strategies.

## 1. Introduction

Paediatric dentistry is a professional discipline that faces unique challenges in both clinical practice and ethics [1]. One of the most difficult areas is the management of pain, anxiety, and fear in children, which influences their perception of and relationship with the dentist throughout life [2]. The experience of pain during dental treatments clearly affects a child’s cooperation, shapes their perception of health professionals, and impacts long-term oral health [3,4]. Ethical principles focus on respecting autonomy and free will (appropriate to the child’s age), performing dental procedures with minimal pain, and upholding the principles of non-maleficence and justice, which are fundamental in paediatric care [5]. All this evidence suggests that the ethics of pain management in children is highly complex and involves the dentist’s approach, the child’s perception, and the parent’s subjective observation [6,7].

Despite the well-defined importance of these issues in the published literature, there is limited evidence from Kosovo related to paediatric dental pain management and its evaluation as experienced and perceived by both children and parents within an ethical framework. In particular, there is a lack of structured data assessing communication practices, informed consent processes, and parental involvement in paediatric dental care. Indeed, this indicates a significant evidence gap regarding how ethical principles are translated and interpreted into clinical practice in paediatric dentistry settings in Kosovo.

In dental health institutions, there are no approved guidelines for the management of pain in children, nor a uniform approach to ethical communication regarding pain management in children. This situation can negatively affect the perceptions of both children and parents, lead to refusal of future treatments, and generate general anxiety. Under such circumstances, there is no systematic feedback on the implementation of measures, ethical practices, and pain management strategies in paediatric dentistry in Kosovo, nor on how they are perceived by Kosovar children and parents. We consider that communication between parents and the dentist, parents’ perceptions of their children’s pain, and children’s reflections on pain experienced during dental treatments are important in defining the concept of ethics in dental practice, and we hypothesise that communication practices and the integration of ethical principles in paediatric dental care, particularly in relation to pain management and informed consent, are suboptimal in Kosovo. The results of this study will provide evidence for the management and implementation of ethical standards in paediatric dentistry in Kosovo, and may help improve clinical communication and guide future policy development in this field.

The objective of this study was to evaluate children’s experiences of dental pain and to assess parental perceptions of ethical aspects of paediatric dental care, including communication, informed consent, and pain management practices. In this context, the term “level of ethical communication” refers to the extent to which dental professionals apply ethical principles, particularly autonomy, beneficence, and non-maleficence, in their interactions with children and parents during dental clinical care. It specifically includes observable communication behaviours such as the clarity and completeness of information provided, age-appropriate explanations to the child, active listening to both child and parent concerns, transparency regarding procedures and pain expectations, and the involvement of parents and children in shared decision-making processes.

This survey therefore aims to analyse children’s experiences and parents’ perceptions of dental pain from the perspective of ethical principle implementation, the quality and behavioural expression of ethical communication, and informed consent practices related to paediatric dental services in Kosovo. Understanding these dimensions is important for identifying gaps between ethical standards and clinical practice and for improving the quality of paediatric dental care. The findings may support the development of more structured approaches to communication and pain management and contribute to strengthening trust between parents, children, and dental professionals.

## 2. Methodology

### 2.1. Study Design

This study employed a cross-sectional methodology. The main objective was to collect and analyse data to assess children’s experiences and parents’ perceptions regarding pain, as well as to evaluate the impact of implementing ethical aspects of care in paediatric dentistry in Kosovo.

### 2.2. Ethical Consideration

This research was approved by the Ethics Committee of the Kosovo Dentistry Association, reference number 945, dated 24 December 2025. The Ethics Committee also approved the content of the written informed consent forms for children and their parents regarding voluntary participation in the survey.

This study was conducted in accordance with the ethical principles of autonomy, beneficence, and non-maleficence, which were integrated into the study design and data collection process. Autonomy was ensured through informed parental consent and child assent, with voluntary participation and the right to withdraw at any time. Beneficence was demonstrated by the study’s aim to improve paediatric dental care by enhancing the understanding of pain, communication, and ethical practice. Non-maleficence was maintained by avoiding interference with clinical treatment and by assessing pain experiences without introducing additional risk or procedures. Ethical approval and consent procedures were ensured by approval from the Ethics Committee of the Kosovo Dentistry Association, which enabled us to uphold these principles, ensuring confidentiality, voluntary participation, and participant protection throughout the study.

### 2.3. Risks and Benefits

The research poses no additional risk to participants, as it does not interfere with clinical treatment.

Benefits include improving ethical and pain management practices in paediatric dentistry.

### 2.4. Confidentiality and Data Protection

The data has been encrypted and does not contain any identifying information. Access to the data will be limited to the research team only. The data will be stored securely for a limited period of time.

### 2.5. Right to Withdraw

Participants had the right to withdraw from the research at any time without any consequences for the child’s dental treatment.

### 2.6. Settings

To ensure comprehensiveness and to eliminate potential differences between settings, the study was conducted at the University Clinical Dental Centre of Kosovo (UCDCK) and the Private Dental Clinic “Dent-Fix” in Prishtina, to evaluate experiences in both public and private dental care.

### 2.7. Participants

This study involved 116 children with their respective parents or legal guardians. Participants were selected to meet the following study criteria:

Inclusion Criteria:

Child Cohort: Children aged 6–12 years undergoing routine dental treatments.

Parent/Guardian Cohort: Legal parents or guardians of the participating children who could provide informed consent.

Exclusion Criteria:

Children with severe cognitive or communication impairments that prevented understanding of the procedure, and parents or guardians who were unable to complete the survey due to language barriers or other constraints.

### 2.8. Data Collection Instruments

The questionnaire used in this study was standardised through literature-based development, expert review, and pilot testing. For research purposes, we conducted a comprehensive review of the relevant literature in paediatric dentistry, pain management, and dental ethics [8]. The use of the Wong–Baker Faces Pain Rating Scale for assessing pain in children strengthens the reliability of the primary outcome measurement [9].

To enhance content validity, the initial draft of the instrument was reviewed by experts in paediatric dentistry at UDCCK, and the questionnaire was refined based on their feedback. Prior to the main data collection, the instrument was pilot tested on a small sample of participants (*n* = 10) to assess comprehensibility, clarity of wording, and feasibility in the clinical setting. Minor modifications were made to improve structure and readability.

To ensure that parents accurately understood the survey items in the “Parent–Dentist Communication” section, several methodological measures were implemented during questionnaire administration. Before data collection, the questionnaire was reviewed for clarity and adapted using simple, understandable language appropriate for the local population. A pilot evaluation was also conducted to identify potentially unclear or ambiguous questions.

During data collection, participants received verbal explanations about the purpose and meaning of the questionnaire items when necessary. The questionnaires were administered in the presence of trained researchers, who were available to clarify any uncertainties without influencing participants’ responses. Parents were encouraged to ask questions if any item was unclear before completing the survey. During data collection, participants received verbal explanations regarding the purpose and meaning of the questionnaire items when needed. The questionnaires were administered in the presence of trained researchers who were available to clarify any uncertainties without influencing participants’ responses. Parents were encouraged to ask questions if any item was unclear before completing the survey. Data were collected through semi-structured interviews with children and their parents. These interviews gathered information regarding communication, informed consent, and satisfaction. The questionnaire consisted of two parts:

PART A: Child Interview (5–12 years), with four sections: Section 1: General experience; Section 2: Pain; Section 3: Help and communication; Section 4: Emotional experience.

PART B: Parent/Guardian Questionnaire, with four sections: Section 1: Demographic data; Section 2: Information and consent; Section 3: Ethical attitudes; Section 4: Trust and satisfaction.

### 2.9. Research Variables

The main outcomes were children’s pain experience during dental procedures; parents’ perception of dental services; implementation of ethical principles; and parents’ satisfaction with pain management.

The independent variables were child-related demographic factors (age, gender, dental experience); treatment-related factors (type of dental procedure, pain management method used); parent/guardian-related factors (education level, prior experience with dental care for children, consent provided); and clinic-related factors (communication style of dental staff, implementation of ethical protocols in the clinic).

### 2.10. Treatment of Bias

To reduce bias, several measures were taken: standardised questionnaires were assessed multiple times before use with participants to minimise interviewer influence; anonymity and confidentiality were assured to reduce social desirability bias; and staff were trained on neutral phrasing and consistent instructions to participants.

### 2.11. Sample Size

The sample size of 116 respondents was considered sufficient according to the sample size calculation for this survey. The sample size was calculated using the Chi-square goodness-of-fit framework with a medium effect size (Cohen’s w = 0.3), 95% confidence (α = 0.05), and 80% study power (1 − β = 0.80), using the following formula:

$$N=\frac{\left(\chi_{1-\alpha ,df}^{2}+\chi_{1-\beta ,df}^{2}\right)}{w^{2}}$$

where confidence level (α) was set at 0.05 (95% confidence); statistical power (1 − β) at 0.80 (80% power); effect size (Cohen’s w) at 0.3, representing a medium effect; and degree of freedom (df) = k − 1.

According to these parameters, the minimum number of participants needed was 98; therefore, the cohort of 116 respondents exceeded this threshold and ensured robust interpretation of meaningful differences in categorical responses.

### 2.12. Statistical Analysis

The data collected were categorised by region, age group, and years of experience, and were analysed using descriptive statistics presented as percentages, frequencies, means, and standard deviations. For inferential analysis, the Chi-square test was used to compare nominal groups, the *t*-test for independent samples was used to compare group means, and the Spearman/Pearson correlation was used to assess possible correlations between experience and self-perception for the difficult patient.

Data were entered and analysed using Microsoft Excel 365 (Office 365). For demographic parameters, we used descriptive statistics, including frequencies, percentages, means, and standard deviations. For other study variables, such as children’s experiences, parents’ perceptions, and reported pain levels, we used the Chi-square goodness-of-fit test to examine differences. The Chi-square test or Fisher’s exact test was used to examine associations between independent variables and main outcomes, where appropriate. A significance level of *p* < 0.05 was considered statistically significant. Descriptive statistics were applied to summarise demographic and questionnaire data, while Chi-square or Fisher’s exact tests were used for categorical variables, *t*-tests for continuous variables, and correlation analyses for predefined associations between selected variables.

## 3. Results

A total of 116 children were included in this study, each accompanied by one parent during their dental visit. The demographic characteristics of the study population are presented in Table 1.

In Table 1, values are presented as frequencies and percentages of study participants. The table summarises the demographic characteristics of children and accompanying parents included in the study.

The sample of 116 children was relatively gender-balanced (50% female and males). Most of them were in the 11–12-year age group (50.9%) and lived in an urban residence (74.1%). The dominant category of parenteral education was secondary school (46.6%), and most visits were performed in the public health setting (94%).

The findings presented in Table 2 show that the majority of children were informed by their parents that they would visit the dentist (97.4%, χ^2^ = 209.4, *p* < 0.001), and their parents explained the reason for the visit (94.8%, χ^2^ = 185.0, *p* < 0.001). The vast majority of children reported a positive feeling and experience during the dental visit (51.8% very good and 40.5% good). The dental visit was accompanied by continuous care and inquiries about the children’s feelings during the visit (94%, χ^2^ = 173.2, *p* < 0.001). However, the most frequently reported emotion was fear (48.3%), followed by trust (25.9%) and calmness (19.0%). Nevertheless, 90.5% of children stated that they would like to visit the same dentist again.

The data in Table 3 show that most children (94.0%) stated that the dentist had explained the procedure to them in advance. The factor that helped children most to reduce fear was communication with the dentist during the procedure (61.2%), followed by the presence of a parent (29.3%). Most children did not experience any pain (45.8%) or experienced only a little pain (51.8%) during the visit; only a very small number experienced mild or a lot of pain (1.6% and 0.8%). Among those who reported pain (n = 63), most children emphasised that the pain was more pronounced during the needle puncture (39.7%), while a smaller proportion experienced pain during the procedure (13.7%) and only 0.8% after the visit (χ^2^ = 119.85, *p* < 0.001). In 93.1% of cases, children reported that they told the dentist if they felt pain during the procedure. Statistical tests showed that the dentist explaining the procedure to the child during the visit, the child experiencing no pain or little pain, communication with the dentist during the procedure, and the presence of a parent were most helpful in reducing fear.

Table 4 shows that the dentist explained the procedures to the child in 74.1% of cases, while only 8.6% of parents reported that the dentist did not do so. In 88.8% of cases, the dentist discussed potential experiences the child might have during procedures, reflecting a good standard of parent–dentist communication. More than half of the parents (51.7%) declared that written informed consent was obtained, while 36.2% reported verbal informed consent, showing a significant difference (χ^2^(2) = 31.52, *p* < 0.001) in the formalisation of informed consent. Regarding the perception of whether pain is unavoidable, parents’ responses were more balanced: 35.3% answered yes, 37.9% answered no, and 26.8% were unsure. Furthermore, the majority of parents reported that their children experienced no pain (44.8%) or minor pain (43.1%), while fewer cases reported mild (9.5%) or intense pain (2.6%).

Table 5 presents parents’ attitudes related to various ethical aspects in paediatric dentistry, showing that most respondents had a positive ethical approach, indicating a high level of awareness among participants regarding appropriate pain management for dental pain in children. The majority of parents (77.6%) agreed with drug treatment for dental pain, despite being aware of the potential side effects, with a significant difference between categories (χ^2^ = 18.62, df = 3, *p* = 0.0003). Parents reported feeling free to refuse treatment in 89.7% of cases, showing a statistically significant difference (χ^2^ = 72.55, df = 1, *p* < 0.001). A total of 94.8% of parents stated they would seek dental services, while only 5.2% would refuse if their child refused to see the dentist (χ^2^ = 90.76, df = 1, *p* < 0.001). In 81.9% of cases, parents reported that they respect their children’s wishes to refuse dental procedures (χ^2^ = 52.14, df = 1, *p* < 0.001). Furthermore, 96.6% of parents were satisfied with dental services, 3.4% were neutral, and none were dissatisfied (χ^2^ = 58.33, df = 2, *p* < 0.001). Additionally, 96.6% would recommend the dental services, while 3.4% would not (χ^2^ = 101.38, df = 1, *p* < 0.001). Regarding the need for legal guidelines for children’s pain management in dental services, 79.3% agreed, 17.2% were unsure, and only 3.4% were against (χ^2^ = 14.22, df = 3, *p* = 0.027). Written procedures for ethical aspects of pain management were preferred by 77.6% of parents, while 19.0% were unsure and 3.4% were against (χ^2^ = 13.67, df = 3, *p* = 0.033).

## 4. Discussion

This study provides important insights into children’s dental pain experiences and parental perceptions of the ethical aspects of paediatric dental care in Kosovo. Overall, the findings indicate generally positive reported experiences, high levels of satisfaction, and frequent use of communication and consent practices in clinical settings. However, these results should be interpreted within a broader contextual and methodological framework, rather than as direct evidence of fully optimised ethical practice.

The demographic characteristics of the study sample, such as equal gender distribution, reduce potential bias, while the predominance of older children (11–12 years) may reflect more reliable self-reporting of dental experiences. Moreover, self-reporting is considered valuable in dental epidemiological investigations [10]. Most children were accompanied by their mothers, which may have provided emotional comfort but may also have introduced parental influence on children’s responses, potentially affecting the independence of reported experiences. The relatively high educational level of parents (46.6% with secondary education, 29.3% with a bachelor’s degree) may have contributed to better understanding of dental procedures and communication, but may also increase socially desirable responses regarding satisfaction and trust in dental care.

One of the important findings of this study is the coexistence of a high level of fear reported by children (48.3%) alongside a very high level of parenteral satisfaction with dental care (96.6%) and positive overall experience. This apparent contradiction reveals that satisfaction with dental services does not necessarily indicate the absence of emotional distress during dental treatment. This can include procedural anxiety, particularly in paediatric dentistry, where fear is often anticipatory and linked to previous experiences, parental anxiety, or fear of specific procedures such as injections. Similar findings were reported by Bayon et al., who indicated that parental anxiety disorders significantly affect children’s emotional behaviour and cooperation during dental procedures. These findings suggest that parental emotional status may directly impact children’s fear perception and behaviour during dental treatment [11].

Parental preparation before the dental visit appears to play a significant role in shaping children’s experiences. Almost all children reported being informed about the visit (97.4%) and its purpose (94.8%). While this reflects good communication practices, it may also represent parent-driven framing of dental care that emphasises necessity rather than emotional reassurance, which can influence how children perceive the procedure. Parent–child communication is known to reduce uncertainty, but it may also unintentionally reinforce expectations of discomfort depending on how information is delivered. Parent–child communication before a dental visit is considered very important for reducing uncertainty and anxiety in children, enabling psychological preparation, and creating the prerequisites for forming more realistic expectations and increasing cooperation during treatment, as confirmed by Yuan, S. et al. [12].

In Kosovo, parental decision-making is further influenced by sociocultural norms, including strong parental authority, protective attitudes towards children, and a preference for clinician-directed decision-making in medical and dental care. These cultural characteristics may affect how ethical principles are understood and applied in practice, especially in situations involving children’s discomfort or refusal of treatment, and should be evaluated in other surveys. The findings show that children generally reported a positive dental experience (97.4%), which may reflect effective clinical management and child-friendly communication strategies. However, this result should be interpreted with caution, as high satisfaction scores in healthcare settings are often influenced by courtesy bias and the desire to provide socially acceptable responses, particularly in the presence of parents. The literature emphasises that positive early dental experiences are important for long-term oral health behaviour, but they do not always fully eliminate dental anxiety [13,14].

However, the results also show that fear in children remained high (48.3%), although a significant proportion reported positive emotions such as confidence (25.9%) and calmness (19.0%). This indicates that dental fear is a phenomenon that affects children, even when the overall experience is positive, which aligns with findings from other surveys [15].

An important aspect of clinical communication was that 94.0% of children reported the dentist repeatedly asked how they were feeling during the procedure, indicating not only effective communication but also active involvement of the children, with positive effects on establishing an effective doctor–patient relationship. This aligns with the conclusion of Ho, J. et al. [16]. Furthermore, the majority of children (90.5%) stated they would like to visit the same dentist again, reflecting a high level of trust and satisfaction with the care received.

Appropriate communication with the child, using techniques such as explaining dental procedures before their application (94%), continuous questioning of how the child feels (61.2%), involving parents during the dental procedure (29.3%), and using distraction methods (9.5%), was found in our research to be at a satisfactory level. According to the literature, these approaches can also contribute to improving the child’s experience of dental procedures. Children’s responses regarding pain during dental visits were dominated by reports of low pain levels, with most stating they experienced little pain (51.8%) or no pain (45.8%). Only a negligible number reported moderate (1.6%) or high (0.8%) pain. An important finding of this study is that pain is experienced mainly during the administration of the needle injection (39.7%), while much lower percentages reported pain during the treatment itself (13.7%) or immediately after it (0.8%). No cases reported late pain, confirming that the pain is mainly related to a specific moment of the dental procedure. These results are consistent with the existing literature, which also identifies local injection as one of the main factors causing anxiety and discomfort in paediatric patients [17]. Fear of needles and the stinging sensation are often perceived as the most unpleasant aspects of dental treatment, even when the procedure as a whole is not painful [18].

Parental–dental communication regarding procedure explanation (74.1%) and pain discussion (88.8%) is well established, indicating a high level of clinical awareness of the importance of pain-related communication and reflecting professional dental practice [19]. Informed consent (written 51.7% and verbal 36.2%) is mainly formalised and constitutes an essential ethical and legal element in dental healthcare. According to Farisato-Touceda et al., the processes of informed consent and assent in paediatric dentistry are more strongly linked to children’s cognitive development and education than to parental education. Indeed, according to these authors, informed consent and assent in paediatric dentistry should not be viewed merely as administrative procedures, but as dynamic ethical processes linked to children’s cognitive development, communication quality, and meaningful participation in decision-making. Therefore, while consent procedures were frequently reported in our study, the findings do not objectively demonstrate the depth of the children’s understanding or the extent to which assent was genuinely integrated into clinical practice [20]. Parental perception of whether pain is unavoidable for their children remains unclear and not well defined (35.3% indicated yes, 37.9% no, and 26.8% unsure) and should be included in discussions on strategies for ethical paediatric dental practice [21].

In the analysis of data regarding parents’ ethical approaches to pain management in paediatric patients, we found a high level of parental support for managing dental pain in paediatric patients, with 77.6% agreeing with the use of drug management for pain, even though they are aware of the side effects of painkillers. Similar conclusions have been reported in studies showing that parents uphold interventions that relieve pain and improve their child’s comfort during dental care [22]. Furthermore, the results show a high level of parental autonomy in decision-making during dental procedures, with the majority (89.7%) reporting that they feel free to refuse treatment for their children, indicating a good level of health education among parents. However, the ethical implications of this finding are complex and should be interpreted carefully. Parents may report respecting a child’s refusal while simultaneously encouraging treatment completion because of concerns regarding oral health outcomes. Consequently, questionnaire responses may reflect idealised ethical attitudes rather than actual behavioural practices during stressful clinical situations. Capurro et al. demonstrated that parental perceptions do not always accurately reflect children’s clinical or emotional conditions, particularly in relation to oral health and treatment experiences. Their findings support the possibility that the parental assessments of low pain intensity and high satisfaction in our study may not fully capture the emotional burden experienced by children during dental procedures [23].

Overall, the findings reflect a high degree of trust in dental procedures and satisfaction (96.6%) with current pain management practices. Moreover, the results show broad support for clear guidelines for dental procedures and emphasise the importance of effective written communication in paediatric care. Paediatric dentists should provide patient-centred care for the children and parents attending their clinics for dental treatment [24].

A key finding is the coexistence of high satisfaction and significant levels of dental fear among children, suggesting that positive clinical encounters do not necessarily eliminate emotional distress. This highlights the multidimensional nature of paediatric dental experiences, where pain, anxiety, communication, and environmental factors interact in complex ways.

The results also indicate that while communication and informed consent practices are commonly reported, their effectiveness may be influenced by subjective perceptions, parental involvement, and sociocultural factors, including strong parental authority and clinician-centred decision-making norms in Kosovo. These factors may shape both children’s responses and parental evaluations of care.

From an ethical perspective, this study suggests that principles such as autonomy, beneficence, and non-maleficence are present in practice but may not always be fully operationalised in a consistent or measurable way. In particular, the relationship between formal consent procedures and true understanding, as well as between communication frequency and emotional reassurance, requires further investigation.

In conclusion, while the findings suggest generally positive perceptions of paediatric dental care, they also reveal important gaps between perceived care quality, emotional experiences, and ethical implementation in practice. Future research should employ more robust methodological designs, validated instruments, and culturally sensitive frameworks to better understand how ethical principles are translated into real-world paediatric dental care and how they influence both pain and anxiety in children.

## 5. Limitations

This study has several methodological limitations that should be considered when interpreting the findings. First, the cross-sectional design does not permit causal inferences between pain experiences, communication, and ethical perceptions. Second, data were based on self-reports from children and parents, which are subjective and may be affected by recall and social desirability bias. Additionally, the absence of a fully standardised paediatric pain scale is a methodological limitation, although age-appropriate response formats were used. Third, the use of convenience sampling limits generalisability, as the sample primarily reflects urban and public dental service users and may not represent rural or private healthcare settings. Fourth, despite standardised procedures, social desirability and parental influence on responses cannot be excluded, particularly for satisfaction and ethical perception items. Fifth, the questionnaire was newly developed and only preliminarily validated, necessitating further large-scale psychometric testing. Finally, the exploratory statistical approach with multiple comparisons may increase the risk of type I error, so findings should be interpreted with caution. Future studies should address these limitations by using longitudinal designs, probability-based sampling methods, standardised validated pain instruments, and more advanced analytical models to strengthen causal inference and external validity.

## Figures and Tables

**Table 1:** The demographic characteristics of the study population.

Variable 1	Category	*n* (%)
Gender	Female	58 (50.0)
	Male	58 (50.0)
Age group (years)	5–7	16 (13.8)
	8–10	41 (35.3)
	11–12	59 (50.9)
Accompanying parent	Mother	66 (56.9)
	Father	50 (43.1)
Parental education	Primary school	18 (15.5)
	Secondary school	54 (46.6)
	Bachelor’s degree	34 (29.3)
	Postgraduate degree	10 (8.6)
Residence	Urban	86 (74.1)
	Rural	30 (25.9)
Dental service setting	Public	109 (94.0)
	Private	7 (6.0)

**Table 2:** Children’s experiences during dental visit.

Study Questions	Category of Answer	*n* (%)	Chi-Square (χ^2^)	df	*p*-Value
Did your parents told you that you will visit your dentist?	Yes	113 (97.4)	209.4	1	<0.001
	No	3 (2.6)			
Did your parents explained to you why you are going to visit your dentist?	Yes	110 (94.8)	185.0	1	<0.001
	No	6 (5.2)			
How did you feel during your visit?	Very good	60 (51.8)	88.7	3	<0.001
	Good	47 (40.5)			
	Neutral	7 (6.0)			
	Bad	2 (1.7)			
Did the dentist continuously ask you how you were feeling during the procedure	Yes	109 (94.0)	173.2	1	<0.001
	No	7 (6.0)			
What feeling did you experience most during the visit?	Fear	56 (48.2)	38.3	3	<0.001
	Trust	30 (25.9)			
	Calmness	22 (19.0)			
	Pain	8 (6.9)			
Would you like to visit the same Dentist again?	Yes	105 (90.5)	149.7	2	<0.001
	No	6 (5.2)			
	Don’t know	5 (4.3)			

**Table 3:** Children’s pain experienced during dental visit (N = 116).

Study Questions	Category of Answer	*n* (%)	Chi-Square (χ^2^)	*p*-Value
Did the dentist explained to you what will do prior to intervention?	Yes	109 (94.0)	89.69	<0.001
	No	7 (6.0)		
What helped to you most to do not have fear?	Talkative Dentist	71 (61.2)	99.57	<0.001
	My parents were close to me	34 (29.3)		
	Music/distraction	11 (9.5)		
	Nothing			
Did you experience pain during the visit?	No	53 (45.8)	105.17	<0.001
	A little	60 (51.8)		
	Mild pain	2 (1.6)		
	A lot of pain	1 (0.8)		
If yes, when did you experience pain (n = 63)?	During the needle shot	46 (39.7)	87.64	<0.001
	During the treatment	16 (13.7)		
	Right after the treatment	1 (0.8)		
	Late after the treatment	0 (0.0)		
Did you tell the dentist if you are experiencing pain?	Yes	108 (93.1)	86.21	<0.001
	No	8 (6.9)		

**Table 4:** Parent–dentist communication (N = 116).

Study Questions	Category of Answer	*n* (%)	Chi-Square (χ^2^)	df	*p*-Value
In the beginning, did the dentist explained to you the necessary procedures?	Yes, clearly	86 (74.2)	72.48	2	<0.001
	Yes, partially	20 (17.2)			
	No	10 (8.6)			
Have you discussed about the pain management?	Yes	103 (88.8)	69.67	1	<0.001
	No	13 (11.2)			
Did they require your treatment consent approval?	Yes, in written form	60 (51.7)	31.52	2	<0.001
	Yes, verbally	42 (36.2)			
	No	14 (12.1)			
Do you think that the pain is unavoidable?	Yes	41 (35.3)	3.92	2	0.141
	No	44 (37.9)			
	Not so sure	31 (26.8)			
Based on your perception, how much pain your child has experienced?	None	52 (44.8)	41.83	3	<0.001
	Minor pain	50 (43.1)			
	Mild pain	11 (9.5)			
	Intensive pain	3 (2.6)			

**Table 5:** Parents’ ethical approaches to pain management in paediatric patients.

Study Questions	Category of Answer	*n* (%)	Chi-Square (χ^2^)	df	*p*-Value
The parent opinion if dental pain in children should be treated, even if pain medication may have side effects	Agree	90 (77.6)	18.62	2	0.0003
	I don’t know	20 (17.2)			
	Against it	6 (5.2)			
Do you respect your child’s wish to refuse dental procedures?	Yes	95 (81.9)	52.15	1	<0.001
	No	12 (18.1)			
Do you feel free to ask or refuse a procedure?	Yes	104 (89.7)	72.55	1	<0.001
	No	12 (10.3)			
If your child refuses the dental procedures, do you seek help from the dentist?	Yes	110 (94.8)	90.76	1	<0.001
	No	6 (5.2)			
How much satisfied you are with the way that your child pain was managed?	Satisfied	112 (96.6)	58.32	2	<0.001
	Neutral	4 (3.4)			
	Unsatisfied	0 (0.0)			
Would you recommend this dental service provider for other children?	Yes	112 (96.6)	101.38	1	<0.001
	No	4 (3.4)			
Do you think that the ethical aspects of pain management in children should be regulated by legal guidelines for dental practice	Agree	92 (79.3)	14.2	2	0.027
	I don’t know	20 (17.2)			
	Against it	4 (3.4)			
Do you think that the ethical aspects of pain management in children should be written and accessible to parents in the dental clinic?	Agree	90 (77.6)	13.67	2	0.033
	I don’t know	22 (19.0)			
	Against it	4 (3.4)			

## Data Availability

The data presented in this study are available from the corresponding author upon reasonable request. Access to the data is restricted due to the inclusion of sensitive information related to child participants and in accordance with the requirements of the Ethics Committee.
